# Ligands Binding to Cell Surface Ganglioside GD2 Cause Src-Dependent Activation of N-Methyl-D-Aspartate Receptor Signaling and Changes in Cellular Morphology

**DOI:** 10.1371/journal.pone.0134255

**Published:** 2015-08-07

**Authors:** Wenyong Tong, Mario Maira, Martin Gagnon, H. Uri Saragovi

**Affiliations:** 1 Lady Davis Institute-Jewish General Hospital, McGill University, 3755 Cote St., Catherine, E-535, Montreal, Quebec, Canada; 2 Pharmacology and Therapeutics, McGill University, 3755 Cote St., Catherine, E-535, Montreal, Quebec, Canada; 3 Segal Center for Translational Research, McGill University, 3755 Cote St., Catherine, E-535, Montreal, Quebec, Canada; University of Louisville, UNITED STATES

## Abstract

Ganglioside GD2 is a plasma membrane glycosphinogolipid. In healthy adults it is expressed at low levels, but it is over-expressed in many cancers. For cancer therapy, GD2 is targeted with anti-GD2 monoclonal antibodies (mAbs), and one adverse side effect is severe visceral pain. Pain is not neuropathic, cannot be blocked with morphine, and stops on discontinuation of mAb therapy. Here, we provide evidence that ligand binding to cell surface GD2 induces rapid and transient activation of Src-family kinases, followed by Src-dependent phosphorylation of NMDA-receptor NR2B subunits selectively, activation of Ca^++^ fluxes, production of cAMP, and changes in cellular morphology. These GD2-ligand activated signals differ in kinetics and in pharmacology from activation of the same signals in the same cells by BDNF, the growth factor agonist of the TrkB receptor, suggesting biological specificity. Hence, cell surface GD2 regulates pathways that can be associated with neoplasia and with morphine-intractable pain; and this can explain why expression of GD2 correlates with these two pathologies.

## Introduction

Ganglioside GD2 is a glycosphinogolipid expressed at high levels during embryonic development, but in the adult it is detectable only in a subset of normal peripheral nervous system and in cerebellum [1, 2]. In adult tissues GD2 is re-expressed at very high levels in many types of cancer such as neuroblastoma, small-cell lung carcinoma, and melanoma [3].

There is a poorly understood correlation between oncogenesis and GD2 re-expression. Nonetheless, GD2 is a clinically validated tumor marker and it is targeted using anti-GD2 monoclonal antibodies (mAb) such as IgG3 mAb 3F8 for diagnosis and immunotherapy [4–7].

One undesired side effect of systemic administration of anti-GD2 mAbs in humans is rapid and severe visceral pain [4, 6, 8–11]. Pain is not neuropathic, and resolves rapidly after discontinuation of mAb infusion. Little is known about how anti-GD2 antibodies induce acute pain, but the pain is associated with ectopic activity in afferent C-fibers, and most intriguingly it cannot be blocked by morphine [12, 13].

We asked what biological processes could be mediated by GD2 that could lead to Src-family tyrosine kinases (SFK) activation, to a transformed phenotype, and cause morphine-intractable pain; and hypothesized that these events may share a signaling pathway(s). Indeed, GD2 gangliosides can activate SFKs in lymphoid cells [3, 14, 15], but the actual mechanisms of signal transduction remain unclear. Hence, we focused on Src kinases and on N-Methyl-D-aspartic acid receptors (NMDA-R) for the following reasons: (i) Src is over-expressed/mutated as an oncogene [16]; (ii) NMDA-R, particularly the NR2B subunit, sensitize peripheral nociceptors in visceral pain [17]; and (iii) Src is a known regulator of NMDA-R activity [18].

GD2 can be functionally relevant because it has been implicated in cell-cell recognition, cell matrix attachment, cell growth, and cell differentiation [19]. For our studies, as selective ligands of cell surface GD2 we used anti-GD2 mAb 3F8 and a small peptide termed SS58 [15]. Both mAb 3F8 and peptide SS58 bind to the cell surface carbohydrate moiety of GD2, at the extracellular domain, and cause activation of the intracellular SFK p56^Lck^ in lymphoid cells [15].

Here, we provide evidence in neuronal cell lines that GD2 ligands activate Src with distinct kinetics and pharmacological sensitivity that differs from growth factor-dependent activation, suggesting biological specificity. Activated Src regulates NMDA-R activity (NR2B phosphorylation, Ca++ fluxes, increased cAMP), and changes in neuronal morphology (e.g. neuritic retraction). Hence, cell surface GD2 regulates signals that can be associated with induction of neoplasia and with induction of morphine-intractable pain; and this mechanism can explain why expression of GD2 correlates with these two pathologies.

## Material and Methods

### Cells

SH-SY5Y-TrkB cells are SH-SY5Y human neuroblastoma (ATCC) stably transfected with human TrkB receptor [20] provided by Dr. Nina Schor at University of Rochester. NMB-7 are human neuroblastoma cells. Cells were grown in RPMI 1640 medium (Life Technologies) supplemented with 5% fetal bovine serum, 2 mM glutamine, 10 mM Hepes and penicillin/streptomycin at 37°C in 5% CO_2_ humidified atmosphere. EL4 cells are a mouse lymphoid thymoma derived from C57Bl/6.

Flow cytometry indicated that all these cell lines express similar levels of cell surface ganglioside GD2, and that the SH-SY5Y-TrkB stably expresses the transfected TrkB receptor.

### Cell treatments

Cells (1.5 × 10^6^/well) were added to a 6-well plate and cultured for 20 hrs. Then the media was exchanged to serum free media (SFM, RPMI, 10 mM Hepes, 0.2% bovine serum albumin) and cells were cultured for 2 hrs to reduce baseline activity. The resting cells were then treated for the indicated times as follows: 10 nM anti-GD2 antibody 3F8 [21], control mouse IgG (mIgG, Sigma-Aldrich), 10 μM GD2-binding peptide 58 which is a mimic of mAb 3F8, or 10 μM of a non-binding control peptide 57 [15]. For SH-SY5Y-TrkB cells, BDNF (4 nM) (Millipore) or EGF (20 ng/ml) was used as a positive control. For pharmacological inhibition, the Src kinase inhibitor PP2 (20 μM) (Enzo Life Science), NMDA-R antagonist ketamine (Bioniche Animal Health) (20 μM) or the PKA inhibitor H-89 (Sigma) (20 ng/ml) were added to the resting cells 30 minutes before the indicated treatments.

### Biochemical assays

After treatment, cells were washed with ice-cold PBS (pH 7.4). Then cells were scraped from dishes in NP-40 lysis buffer (20 mM Tris-HCl pH 7.5, 137 mM NaCl, 2 mM EDTA, 1% NONIDET P-40 detergent, protease inhibitor (Roche) and 50 mM sodium orthovanadate) sonicated at 4°C with an ultrasonic processor (Sonics & Materials), cellular debris was removed by centrifugation and protein content in cellular lysates determined by the Bradford method (Bio-Rad). For Fyn immunoprecipitation, whole cell extracts were incubated a Fyn specific mouse monoclonal antibody (Santa-Cruz Biotechnology, sc-434) and Protein G-plus agarose beads (Calbiochem). The samples were then electrophoretically separated on 6% and 8% SDS-PAGE under denaturing and reducing conditions. The resolved proteins were transferred to polyvinylidene difluroride (PVDF) membranes (0.45 μm, Millipore), which were immunoblotted with antibodies directed to the following phosphotyrosine targets: phospho-NMDA receptor 2B subunit (NR2B) pY1472 (Enzo Life Science), phospho-NMDA receptor 2A subunit (NR2A) pY1325 (Abcam), phospho-Src family pY416, phospho-Src pY527, phospho-PLC pY783 (Cell Signaling) and 4G10 anti-phosphotyrosine (Upstate Biotechnology). Blots were visualized using the enhanced chemiluminescence (ECL) system (NEN Life Science Products) and exposure to film (Source). Data were standardized to total protein loaded, by re-probing membranes with antibodies to total Src (Cell Signaling), total NMDA-R (Cell Signaling), anti-β-tubulin III (Sigma-Aldrich), anti-β-actin antibody (Sigma-Aldrich) and from coomassie blue staining of the gels. ImageJ (NIH) was used for quantification of the films.

### Intracellular calcium studies

SH-SY5Y-TrkB cells (6 × 10^4^/100 μl) were plated in 96-well black wall/clear bottom plates (BD Falcon) in regular growth medium and grown overnight. After removing the growth medium, 100 μl of Fluo-8NW dye solution, prepared according to Screen Quest Fluro-8 medium removal calcium assay kit (ABD Bioquest), was added to each well and plates were incubated at 37°C for 1 hr. Positive control ionomycin (5 μM) (Enzo Life Science) and negative control EDTA (5 μM) (Wisent INC) were used to determine the range of the signal. DMSO was the vehicle for PP2. PBS was the vehicle for everything else. The indicated concentrations of ligands were added to the wells, and the assay proceeded for the times shown. Fluorescence detection was done with a filter set of Ex/Em = 490/525 nm using FLUOstar OPTIMA (BMG LABTECH).

### cAMP detection assay

Intracellular cyclic AMP (cAMP) levels were quantified using the direct cAMP enzyme immunoassay kit (Assay Design Inc). Briefly, Human NMB-7 neuroblastoma cells were plated in 96-well culture plates in medium at a density of 9×10^5^ cells/well. Cells were treated with 3F8 (10 nM), SS57 irrelevant control peptide or SS58 GD2 ligand (25 μM). Forskolin (50 μM) was the positive control. After 10 minutes, the treatment was terminated by the aspiration of the culture medium and addition of the lysis buffer included in the kit. cAMP was measured using the nonacetylation assay procedure provided by the manufacturer.

### Morphological assays

Neuroblastoma SH-SY5Y-trkB and NMB-7 cells were grown in 35 mm dishes (Falcon) and cultured in RPMI 1640 medium (Life Technologies) supplemented with 5% fetal bovine serum. 3F8 (50 nM), 3F8+PP2(10 μM), SS58 (10 μM) and SS58+PP2 (10 μM) were added and time-lapse photographs were taken at 100X magnification using a Nikon camera and a microscope adapted with a 37°C chamber.

### FACs experiments

Live 1 x 10^6^ NMB-7 neuroblastoma or EL4 lymphoid cells suspended in FACS buffer (PBS, 0.5% BSA, 0.05% NaN3) were incubated for 15 minutes on ice with anti-GD2 mAb 3F8 (13 nM or 130 nM); with binding controls anti-GM1 cholera toxin B-subunit or with anti-CD45 mAb (50 nM); which yield a high level of immunostaining. Background control is non-binding mouse IgG (Sigma) (25 nM). Cells were washed with FACS buffer, and were freshly acquired (10,000 events/group) in a flow-cytometer (Becton-Dickinson). Data were analyzed using CellQuest software.

### Statistical analysis

One-way and two-way ANOVA with Multiple Comparisons Test were used to compare the different results. Results are reported as significant *P<0.05 and **P<0.01.

## Results

### GD2-ligands alter the phosphorylation of Src kinase

Flow cytometry confirmed that the wild type SH-SY5Y, SH-SY5Y-TrkB and NMB-7 neuroblastoma cells express similar levels of cell surface GD2; and that the transfected SH-SY5Y-TrkB express cell surface TrkB receptors. Using SH-SY5Y-TrkB cells afford a convenient control because the TrkB ligand, the neurotrophin growth factor BDNF, activates the same pathways and can be used to examine the selectivity of signals.

SH-SY5Y-TrkB cells were treated with the indicated test GD2 ligands (3F8 mAb), negative controls (vehicle, or non-binding mouse IgG), or positive controls (BDNF), and the time-dependent changes in phosphorylation of two Src tyrosine residues were quantified. These two residues act as a “rheostat” to Src activity. Phosphorylation of Src-Tyr416 is required for full kinase activation, while phospho-Src-Tyr527 is a residue inhibitory of the kinase activity and its de-phosphorylation is required for full activation.

**Fig 1:**
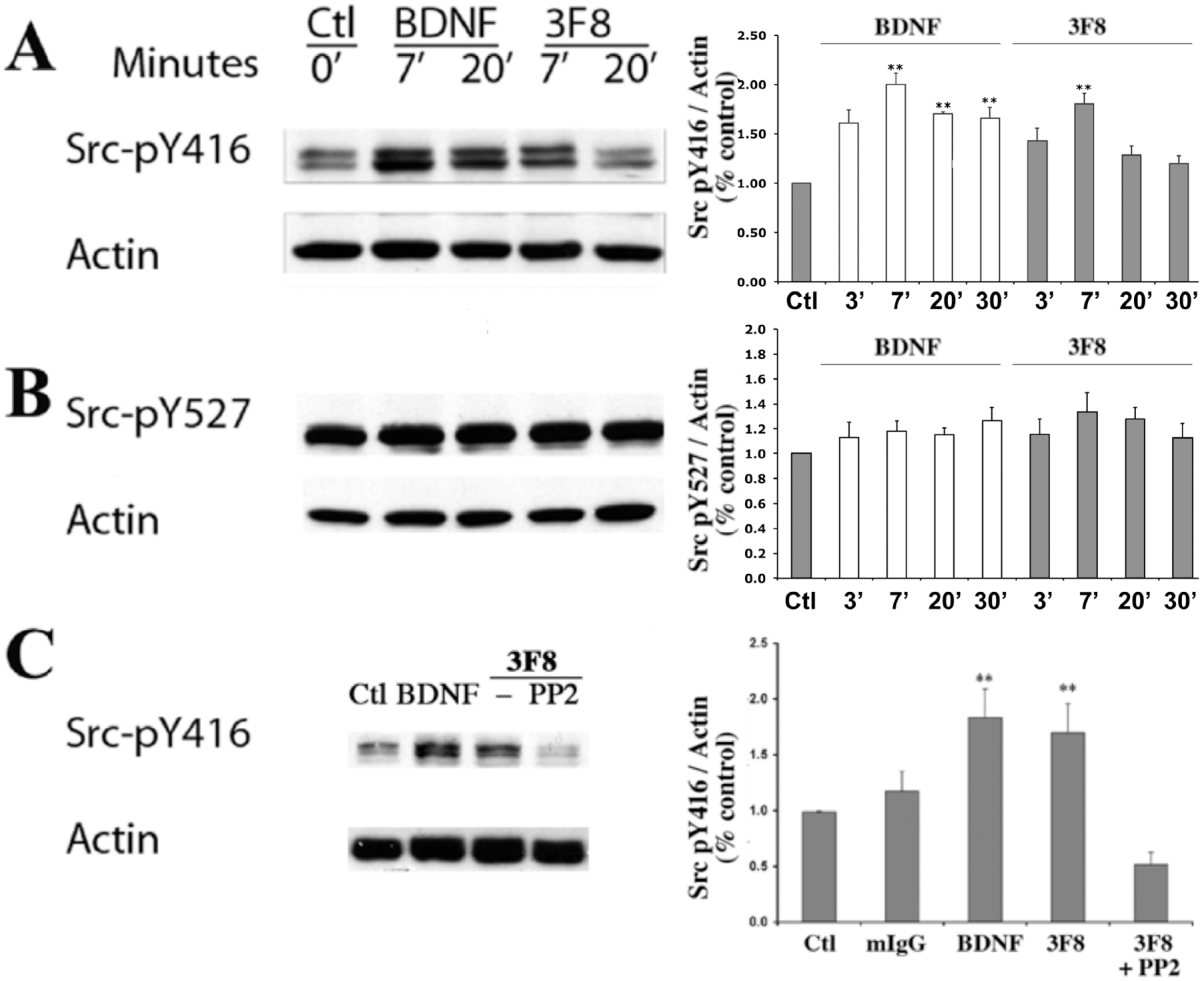
Anti-GD2 mAb 3F8 affects the phosphorylation of Src kinase. SH-SY5Y-TrkB cells were treated with mAb 3F8 (10 nM), control mIgG (10 nM) or positive control BDNF (4 nM) for the indicated times. Western blot analyses of whole cell lysates were done with specific anti-phospho-Src antibodies, and were quantified versus anti-β-actin or β-tubulin III. For quantification, untreated cells were standardized to 1, n = 3–6 independent experiments each in triplicate ± SEM. ** p<0.01 versus control. (A) anti-GD2 antibody 3F8 induces the transient phosphorylation of Src-Tyr^416^. (B) anti-GD2 antibody 3F8 has no effect the phosphorylation of Src-Tyr^527^. (C) Pretreatment of SH-SY5Y-TrkB cells with the Src kinase inhibitor PP2 (20 μM) inhibited 3F8-induced phosphorylation of Src-Tyr^416^.

GD2-ligands caused significant phosphorylation of Src-Tyr416 within 7 min, but the signal was transient and decreased by 20 min (Fig 1A). Control BDNF induced strong phosphorylation of Src-Tyr416 within 7 min, and after 30 min the signal was lower but was still sustained (P< 0.05) (Fig 1A). In pharmacological controls, the GD2 ligand-induced phosphorylation of Src-Tyr416 can be inhibited by the Src inhibitor PP2 (Fig 1C). Interestingly, neither mAb 3F8 nor BDNF significantly altered the baseline phosphorylation of the inhibitory tyrosine, Src-Tyr527 (Fig 1A and 1B). This suggests a differential regulation of the Src rheostat in a neuronal cell line.

In cellular controls using cells devoid of GD2, the GD2-ligands did not affect the phosphorylation of SFK (data not shown). These data indicate that the effect is GD2-ligand specific, dependent on GD2-expression, and that the effect is not restricted to a cell line. Moreover, the effect is not restricted to a neuronal lineage because GD2-ligands activate a Src family member p56^LCK^ in lymphoid cells [15].

### GD2-ligands induce phosphorylation of NR2B via Src kinase

**Fig 2:**
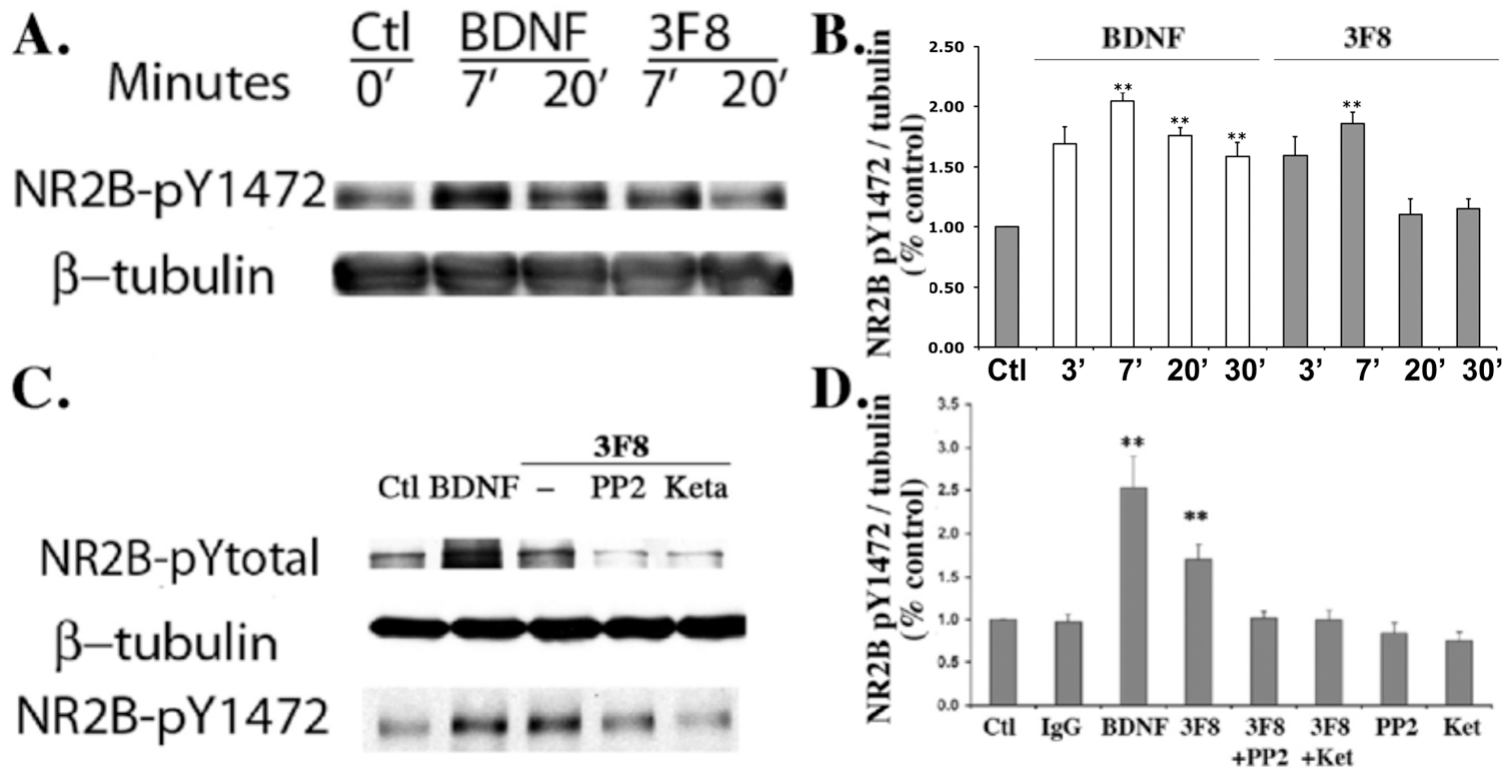
Anti-GD2 mAb 3F8 phosphorylates NR2B via Src kinase. SH-SY5Y-TrkB cells were treated with mAb 3F8 (10 nM), control mIgG (10 nM) or positive control BDNF (4 nM) for the indicated times. Western blot analyses of whole cell lysates were done with specific anti-phospho-NR2B antibodies. Total NR2B levels did not change upon treatment (data not shown). (A) Anti-GD2 antibody 3F8 induces the phosphorylation of NR2B-Tyr^1472^. (B) Data from panel A were standardized to anti-β-actin or β-tubulin III levels, and were quantified versus untreated cells = 100%, n = 3–6 independent experiments each in triplicate ± SEM. ** p<0.01 versus control. (C) Pretreatment of SH-SY5Y-TrkB cells with the Src kinase inhibitor PP2 (20 μM) or the NMDA receptor antagonist ketamine (20 μM) inhibited 3F8-induced phosphorylation of NR2B-Tyr^1472^. (D) Data from panel C were standardized to anti-β-actin or β-tubulin III levels, and were quantified versus untreated cells = 100%, n = 3–6 independent experiments each in triplicate ± SEM. ** p<0.01 versus control.

SH-SY5Y-TrkB cells were treated with ligands or controls as above, and time-dependent changes in phosphorylation of NR2B at Tyr1472 were quantified (Fig 2).

GD2-ligands caused phosphorylation of NR2B-Tyr1472 within 7 min, a signal that was transient and decreased by 20 min (Fig 2A). The positive control BDNF also induced phosphorylation of NR2B-Tyr1472, and the signal was sustained after 30 min, significantly longer than that induced by GD2-ligands (Fig 2A and 2B). Similar results were obtained after GD2-ligand treatment of wild type SH-SY5Y and the NMB-7 neuroblastoma cell line (Figure A in S1 File), indicating that the effect is not exclusive to one neuronal cell line and that the GD2-Src signaling pathway is independent of TrkB expression.

Interestingly, 3F8 (but not BDNF) also increased the phosphorylation of Fyn at Y420 (Figure B in S1 File), suggesting that other SFKs are also regulated by this signaling pathway. However, while activated phospho-Fyn can increase the phosphorylation of NR2A [18] neither mAb 3F8 (Figure B in S1 File) nor BDNF [22] alter the phosphorylation status of NR2A in this cell line. In sum, the GD2-mediated SFK-activation leading to phosphorylation of NR2B appears to be selective. These notion was tested using pharmacological inhibitors.

**Fig 3:**
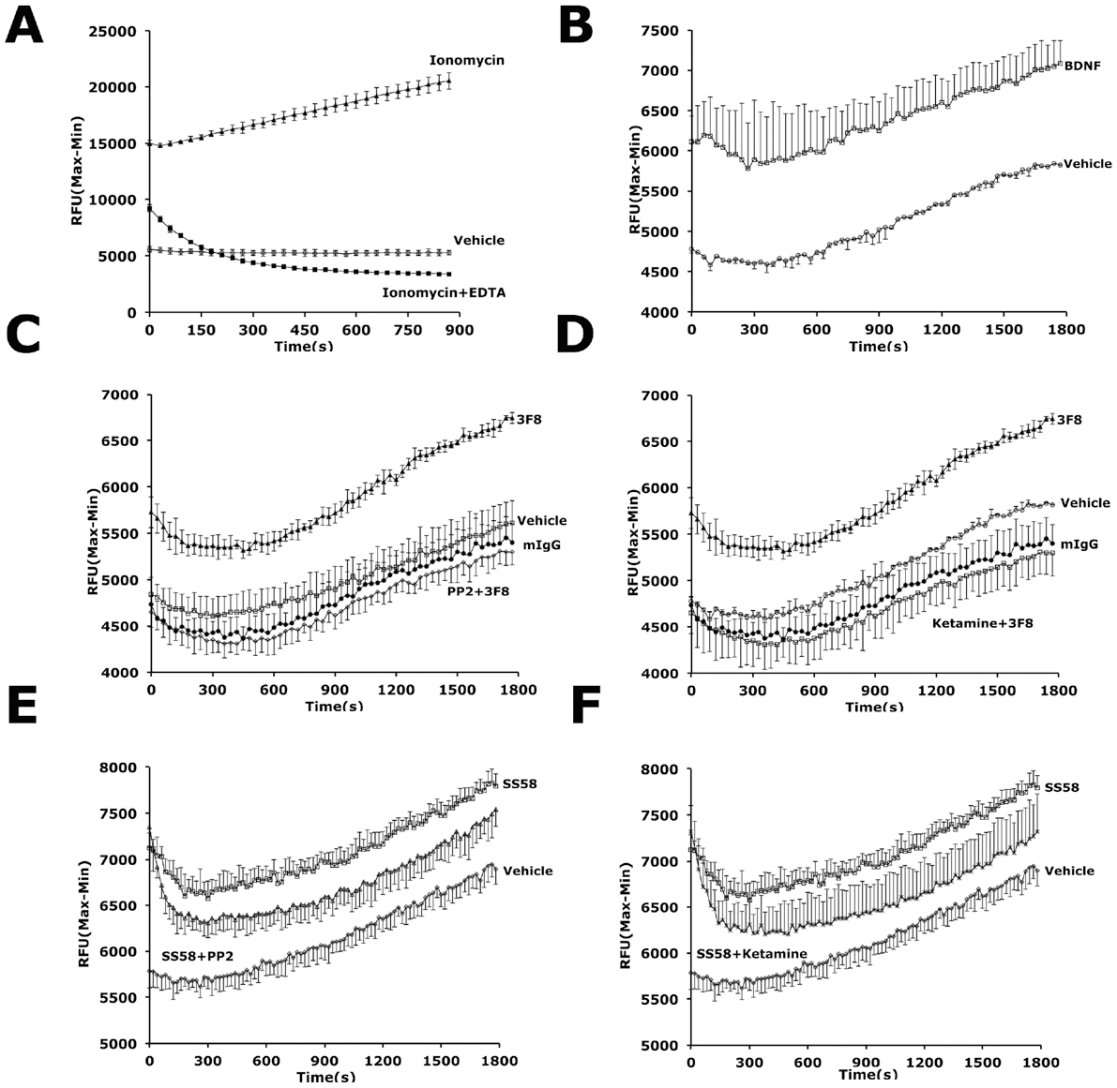
Anti-GD2 ligands regulate calcium fluxes through Src and NMDA-R. SH-SY5Y-TrkB cells loaded with Fluo-8NW dye solution were treated with (**A**) positive control ionomycin (5 μM), or BDNF (4 nM), **(B)** mAb 3F8 (10 nM) (**C, D**) and **(E, F)** SS58 (25 μM). Negative controls buffer or vehicle DMSO, ionomycin plus EDTA to quench Ca^++^, and irrelevant mIgG (10 nM) were also tested. For pharmacological inhibition, the Src kinase inhibitor PP2 (20 μM) or the NMDA-R antagonist ketamine (20 μM) were added 30 minutes before treatment (data shown in **C, D, E and F**, respectively). For Ca^++^ assays, the data are a representative result of 4 independent experiments each done in quadruplicate ± SD.

GD2-ligand phosphorylation of NR2B-Tyr1472 is inhibited by the Src kinase inhibitor PP2 and by the NMDA-R antagonist ketamine (Fig 2C), while the baseline NR2B phosphorylation (in the absence of GD2-ligand) was unaffected by treatment with PP2 or by ketamine (Fig 2D). In contrast, notably, BDNF-induced phosphorylation of NR2B-Tyr1472 and other BDNF signals are not blocked by treatment with PP2 or with ketamine suggesting different mechanisms (data not shown, see related data in Fig 3B and Figure C in S1 File). Thus, the signals activated by the TrkB ligand (BDNF) or the GD2 ligands (3F8 or SS58) activate NR2B with distinct kinetics and sensitivity to pharmacological inhibitors.

### GD2-ligands increase intracellular calcium

To further confirm that GD2-ligands acting through Src activate NMDA-R, Ca^++^ flux was studied in SH-SY5Y-TrkB cells. The ionophore ionomycin was used to calibrate maximal Ca^++^ influx, and ionomycin-effects can be blocked with EDTA (Fig 3A).

The TrkB agonist BDNF (Fig 3B), and the GD2 ligands mAb 3F8 (Fig 3C and 3D), and SS58 (Fig 3E and 3F) can significantly increase the intracellular calcium concentration. The kinetics and the range of the Ca^++^ flux activation were the same for all ligands. Since increased Ca^++^ fluxes could increase adenyl cyclase and PKA activities, which in turn could inhibit Src activation by increasing Csk activity (i.e. phosphorylation of the Src inhibitory site), we tested whether GD2 mAb 3F8 signals in a PKA-dependent manner to increase Src activity. Pre-treatment of SH-SY5Y-TrkB cells with the PKA inhibitor H-89 did not prevent 3F8-dependent increase in Src and NR2B activation, although it did reduce the basal phosphorylation of both (Figure D in S1 File).

### Selectivity of Src-mediated signals induced by GD2-ligands

The Ca^++^ flux induced by GD2-ligands can be blocked by PP2 (Fig 3C and 3E) or by ketamine (Fig 3D and 3F). In contrast, Ca^++^ fluxes induced by BDNF are not blocked by PP2 or by ketamine (Figure C in S1 File). Thus the Ca^++^ flux mechanisms appear to be different for GD2 ligands and BDNF.

**Fig 4:**
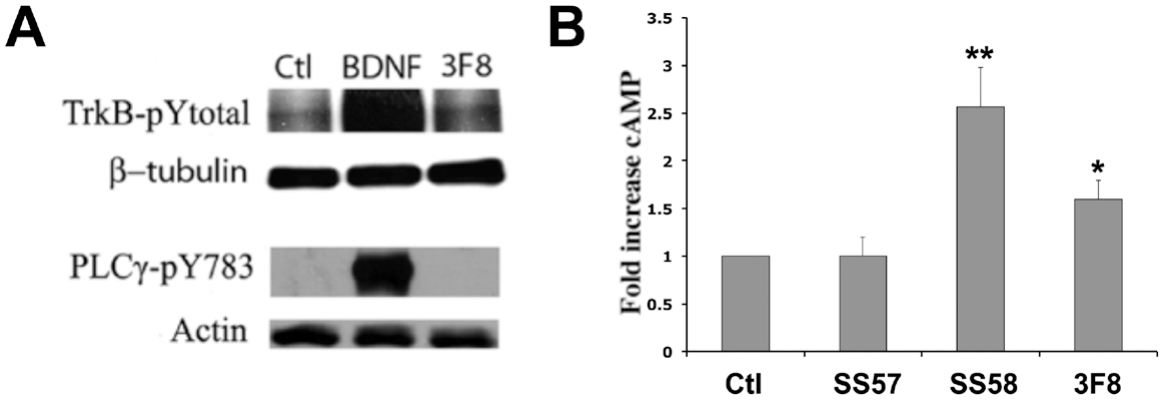
GD2-binding ligands not activate PLCγ, but increase intracellular cAMP. (**A**) Whole cell SH-SY5Y-TrkB lysates were studied by Western blot after treatment with mAb 3F8 (10 nM), negative control mIgG (10nM) or positive control BDNF (4 nM). Only BNDF induces phospho-TrkB and PLCγ-Tyr^783^. (**B**) Anti-GD2 ligands regulate intracellular cAMP. Human NMB-7 neuroblastoma cells were treated with mAb 3F8 (10 nM) or control mIgG (10 nM), peptide SS58 (10 μM) or control peptide SS57 (10 μM). MAb 3F8 and peptide SS58 cause an increase of intracellular cAMP concentration significantly over controls. n = 3–8 independent experiments each in triplicate ± SEM.

Another difference between the Src-signals activated by GD2 ligands and BDNF is that mAb 3F8 did not induce the phosphorylation of PLCγ-Tyr783 (Fig 4A), while BDNF does activate PLCγ. Because GD2 ligands did not activate PLCγ, Ca^++^ fluxes are likely not from internal stores. In addition, while Src activated via GPCR agonists indirectly causes TrkB phosphorylation o [23, 24], the Src-activated via GD2 ligands could not cause TrkB phosphorylation (Fig 4A). These data further substantiate significant differences between GD2-induced Src signaling *versus* GPCR and TrkB-induced Src signaling.

### GD2-ligands increase intracellular cAMP

GD2-ligands increase the intracellular cAMP concentration. Both mAb 3F8 and peptide SS58 significantly increased cAMP levels, but their corresponding negative controls mIgG and a non-binding peptide SS57 did not increase cAMP (Fig 4B). These data are consistent with GD2-ligands enhancing NMDA-R activity and Ca^++^ fluxes.

### Morphological changes in cells treated with GD2 ligands

**Fig 5:**
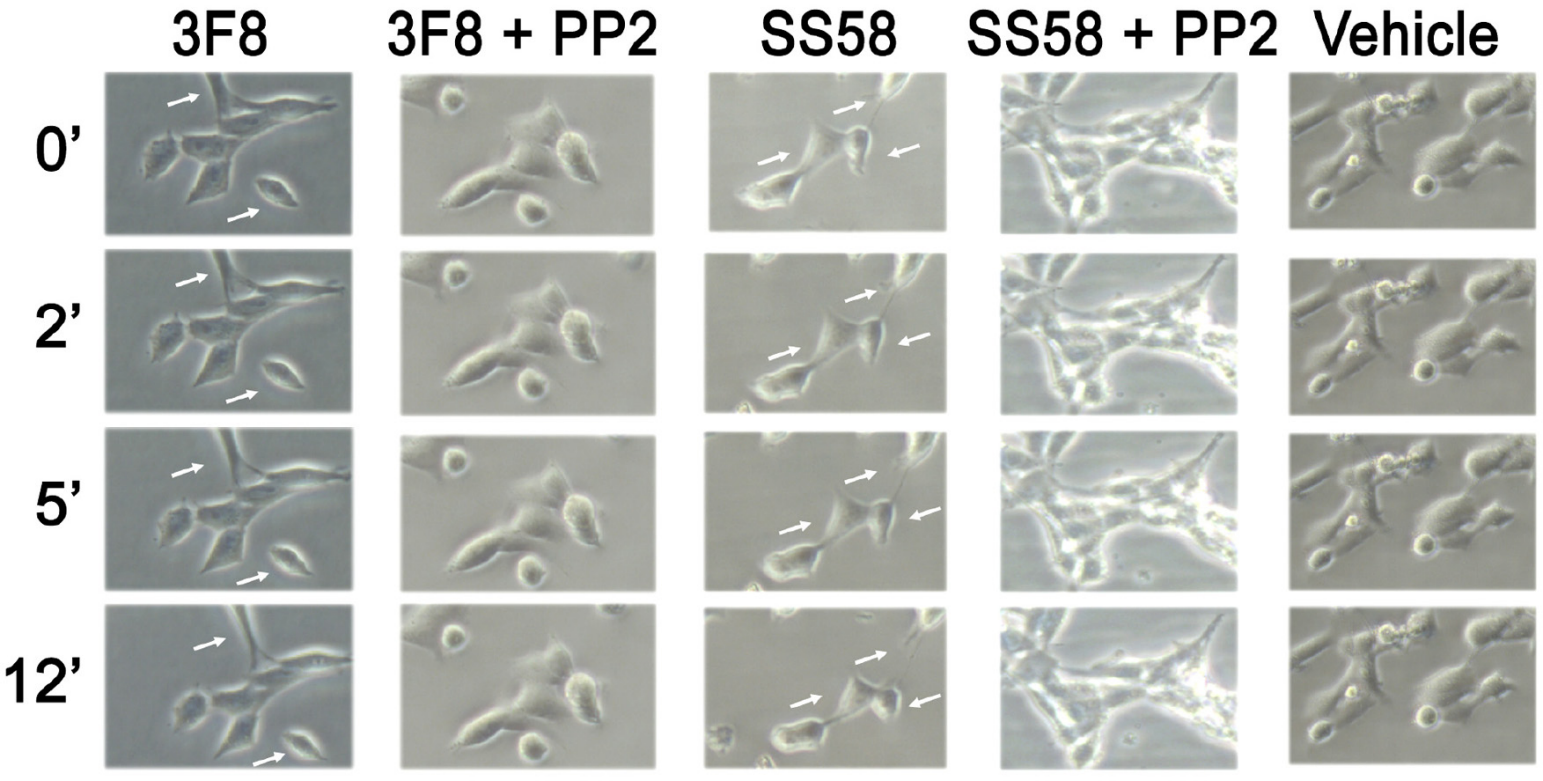
Morphological changes in neuroblastoma cells treated with GD2 ligands. Adherent SY5Y-trkB cells grown in complete media were treated with 3F8 (50 nM) or SS58 (10 μM) with or without PP2. Time-lapse photographs were taken at 100X magnification at the indicated times of incubation: 0’ (control starting time), 2’, 5’, 12’. Cells change in morphology over time.

Induction of GD2 Ca^++^ influxes and elevated intracellular cAMP by GD2 ligands can cause effects on cellular morphology. This was tested in the SY5Y-trkB neuroblastoma cell line (Fig 5), the NMB-7 neuroblastoma cell line (Figure E in S1 File) and the EL4 lymphoid cell line expressing GD2 on the cell surface (Figure F in S1 File).

SY5Y-trkB and NMB-7 cells grow adhered to the plastic dish. Addition of 3F8 mAb or peptide SS58 to the media caused significant morphological changes characterized by rounding/swelling of cells and retraction of their projections. The effect was nearly maximal within 5 minutes (Fig 5 and Figure E in S1 File). The PP2 Src inhibitor can prevent the morphological changes induced by 3F8 or peptide SS58. In ligand controls, no morphological changes were seen when non-binding peptide SS57 or mouse IgG were added to media (data not shown).

In EL4 cells treatment with mAb 3F8 or with peptide SS58 has already been reported to induce Src phosphorylation [15] analogous to what is shown here for neuronal cells. Because EL4 cells grow in suspension, it is possible to study their morphology quantitatively by FACScan. Forward Scatter (FSC) is a measure of cell size/volume and Side Scatter (SSC) is a measure of cellular density/complexity. Binding of 3F8 mAb caused a ~100% increase in cellular volume, and ~100% increase in density/complexity (Figure F in S1 File) in >20 independent experiments.

In the experiment shown, control cells treated with irrelevant IgG, cholera toxin-B subunit binding to GM1, or with anti-CD45 mAbs had an identical size with average FSC of 184 and average SSC of 163 (Figure F in S1 File). After treatment for 15 min with GD2 ligand 3F8 mAb the FSC and SSC increased significantly, and the effect was dose dependent (Figure F in S1 File). In the low dose treatment (13 nM) the FSC increased to an average of 266 and the SSC increased to an average of 296. In the high dose treatment (130 nM) the FSC increased to an average of 295 and the SSC had an average of 440 (Table A in S1 File). Indeed the major morphological changes are detected in the cells with the highest levels of cell surface GD2 staining (Figure F in S1 File). The SSC/FSC data for the cells treated with 3F8 mAb is shown as a function of the levels of fluorescence, and demonstrates that the effect on morphology is GD2-ligand dependent.

In controls for cellular specificity, there were no morphological changes by treatment of an EL4 cell variant that lacks GD2 on the surface (data no shown), indicating that the effect is GD2-dependent.

## Discussion

We demonstrate that ligands of GD2 activate Src-family kinases Src and Fyn with transient kinetics in neuronal cell lines, resulting in phosphorylated NR2B (but not NR2A), leading to biological signals. We [15] and others [3, 14] previously showed that ligands of GD2 can activate a Src-family member p65^LCK^ in lymphoid cells. As well, recently it has been shown in melanoma cells that GD3 expression is involved in activating YES kinase, without affecting Src or Fyn activity [25]. Thus, a ganglioside can regulate the activity of Src kinases, and while regulation is not limited to a cell lineage, the signaling selectivity of gangliosides may be contextual on cell lineage. In addition, artificial ligands of gangliosides (such as 3F8 mAb or SS58) regulate the GD2-mediated control of Src kinases, with a high degree of selectivity. Conceivably, there may be endogenous ligands of gangliosides [26] that potentially could endogenously regulate the GD2-mediated control of Src kinases.

In neuronal cell lines SY5Y, SY5Y-TrkB, and NMB-7, SFK activation by GD2-ligands activates NMDA-R (phosphorylated NR2B), causing increased Ca^++^ influx and intracellular cAMP concentrations. The GD2-ligand activated signals are Src-dependent (e.g. blocked by PP2) and NMDA-R-dependent (e.g. blocked by ketamine). Morever, the GD2-mediated mechanisms appear to be distinct from Src signals activated by BDNF through TrkB.

The activity of Src is regulated by a “rheostat” phosphorylation at two tyrosine residues: the “activating” Src-Tyr416 and the “inhibitory” Src-Tyr527 [27, 28]. Phosphorylation of Src-Tyr416 is required for Src kinase activation, and it is carried out by the Src kinase itself (auto-phosphorylation). The phosphorylation of Src-Tyr527, repressive of Src kinase, is mediated by the Csk kinase. GD2-ligands, as well as BDNF, increased phosphorylation of Src-Tyr416; without detectable changes in phospho-Src-Tyr527 levels compared to untreated control cells. This could be interpreted as a Csk kinase-insensitive Src-activation, and was demonstrated using the PKA inhibitor H-89. PKA is a Csk activator [18] leading to decreased Src activity, but inhibition of PKA had no effect on GD2-mediated signals.

GD2-ligands binding to cell surface GD2 activate Src with kinetics and biology distinct from BDNF-TrkB, suggesting specificity. Additionally it is noteworthy that the GD2-mediated signals differ from BDNF-TrkB in their sensitivity to pharmacological inhibitors. Moreover, GD2-ligands did not cause TrkB phosphorylation, even though GD2-ligands activate Src (this paper), and activated Src is known to cross-phosphorylate TrkB [23]. Lack of TrkB-phosphorylation by Src that was activated via GD2-ligands could be due to cellular compartmentalization. Signals activated by GD2 ligands appear to target the cell surface pool of client receptors (e.g. phospho-NMDA-R can be blocked by ketamine). Hence, GD2 “activation” likely involves cell surface rafts or morphological changes [29–31]. In contrast, ligands of GPCRs can activate TrkB *via* Src but this occurs mainly at intracellular TrkB receptors [23, 24].

The BNDF-induced phospho-TrkB causes phosphorylation of Src. Activated phospho-Src then modulates phospho-NR2B/Ca^++^. These signals serve as positive controls because they have been documented before [22, 32]. In addition to serving as control in our experiments, we also show novel data that BNDF phosphorylation of NR2B/Ca^++^ is not blocked by PP2 or by ketamine. We posit three possible explanations. One is that BDNF can promote NMDA-R phosphorylation via SFK-dependent and SFK-independent ways [33, 34]. A second is that BDNF induces a PLC-γ/IP3-dependent calcium store depletion, which causes non-selective cation influx through other ion channels [35]. A third relates to cellular compartmentalization, where the activity of intracellular TrkB within vesicles may be insensitive to PP2 or ketamine.

**Fig 6:**
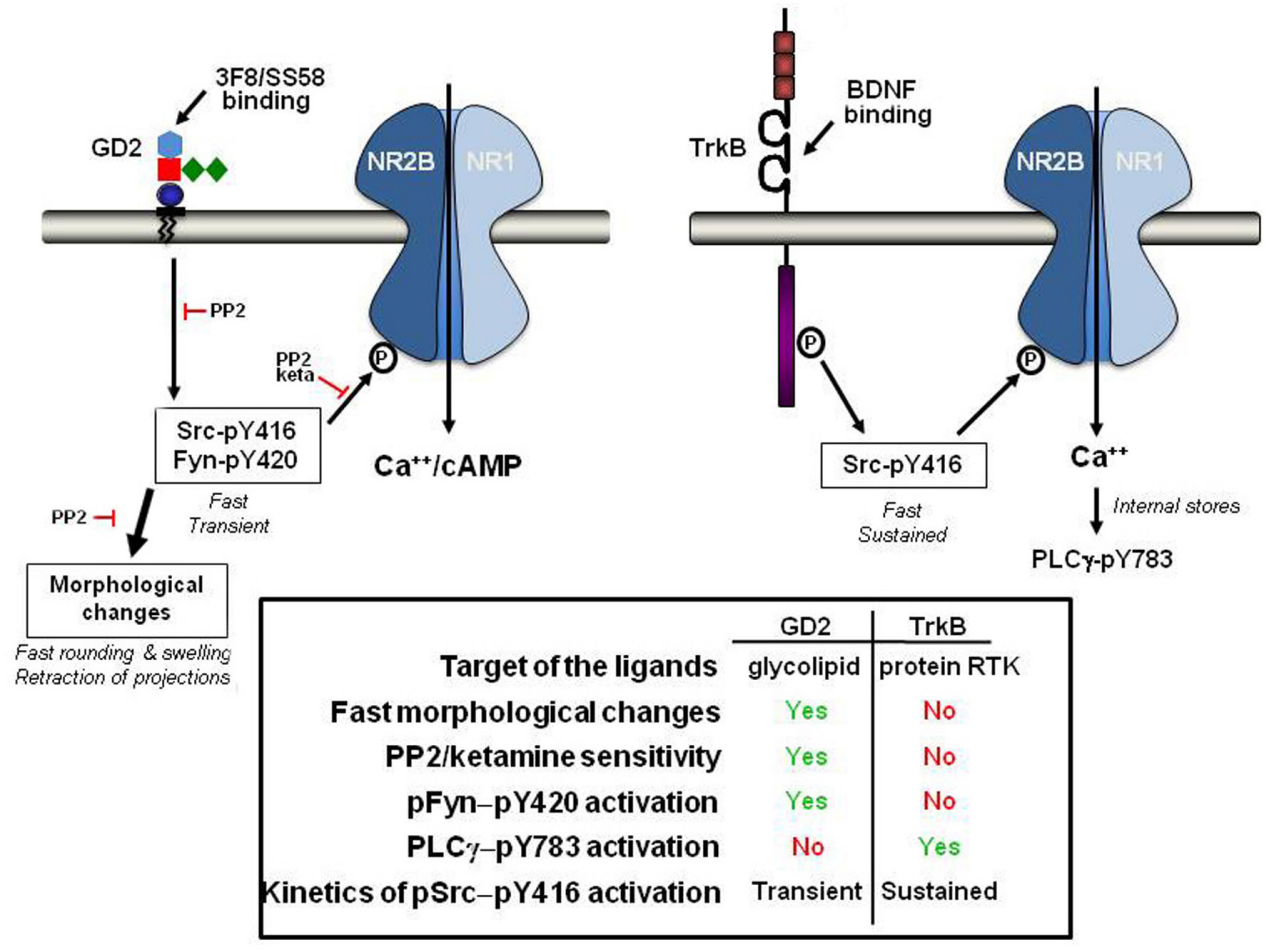
Model of signal pathways activated by GD2 ligands. A comparison of signals activated by GD2 ligands 3F8 or SS58, compared *versus* TrkB ligand BDNF. GD2 (a glycolipid with 5 sugars) and TrkB are not to scale.

Together, our data suggest that GD2 ligands induce phospho-Src leading to selective phospho-NR2B signals that promote an increase of Ca^++^ influx and intracellular cAMP, likely through activation of adenylyl cyclase [36]. The GD2 ligands cause significant morphological changes in neurons including retraction of pseudopodia or axons, and detachment from substrate. Pharmacological inhibition with PP2 indicate that morphological changes are Src–dependent events (see Fig 6 for a model).

Activation of NMDA-R and Ca^++^ fluxes by anti-GD2 mAbs may explain the morphine-intractable visceral pain reported in animal studies [9, 12, 13] and in human clinical trials using therapeutic anti-GD2 mAbs [4, 6, 8, 10, 11]. Previously, in vivo studies combined anti-GD2 mAbs with systemic gabapentin, to reverse allodynia in a dose-dependent fashion [37]. Gabapentin is an analgesic and anticonvulsant structurally related to the neurotransmitter gamma-aminobutyric acid (GABA). The mechanism of action of gabapentin is unrelated to GABA or GABA receptors. Gabapentin is thought to bind to the α2δ subunits [38, 39] of the voltage-gated calcium channel in the central nervous system [40], blocking afferent spinal glutamatergic systems acting through the NMDA-R. This is relevant because studies reported that the NR2B-subunit containing NMDA-R may mediate peripheral sensitization and visceral pain [17]. Also Gabapentin blocks the evoked C-fiber activity in severe peripheral inflammation [41] and this could be a mechanism for achieving allodynia during GD2-targeted therapy.

Taken together, the literature and our study indicate that anti-GD2 antibody induced pain implicate NMDA-R activation and it may be a target-mediated side effect. The peripheral administration of ketamine could attenuate allodynia and visceral pain in patients. However, given the adverse side effects of NMDA-R antagonists, it may be more promising to use pharmacological strategies to uncouple Src from NMDA-R [42]. A recent *in vivo* study demonstrated that Ifenprodil attenuated NMDA-induced allodynia mediated by SFK [43]. Hence this drug might be used in conjunction with anti-GD2 cancer therapy.

In summary, we have shown an unexpected role for cell surface GD2 ganglioside in the modulation of phosphorylation and function of NR2B subunits, through the specific regulation of phosphorylation of Src-Tyr416 (but not Src-Tyr527), leading to increased intracellular Ca^++^ and cAMP, and changes in neuronal morphology. Our findings may impact clinically on how anti-GD2 therapy is applied, and in cell biology on how we consider the role of glycosphinogolipids. For future work, we postulate that other glycosphinogolipids, especially tumor-associated gangliosides may carry out analogous activities through other receptors.

## Supporting Information

S1 File Figure A in S1 File; Figure B in S1 File; Figure C in S1 File; Figure D in S1 File; Figure E in S1 File; Figure F in S1 File. (DOC)
