# Supplementary material for: Ligands Binding to Cell Surface Ganglioside GD2 Cause Src-Dependent Activation of N-Methyl-D-Aspartate Receptor Signaling and Changes in Cellular Morphology
Source: PLoS One. 2015 Aug 7;10(8):e0134255. doi: 10.1371/journal.pone.0134255 (PMC4529173; doi:10.1371/journal.pone.0134255)
Supplement: S1 File — (DOC) [file pone.0134255.s001.doc]

**SUPPLEMENTAL FIGURES and TABLE**

**Figure A.** **Anti-GD2 mAb 3F8 phosphorylates NR2B-Tyr^1472^ in SY5Y and NMB-7 cells.**

**Figure B.** **Anti-GD2 mAb 3F8 induces the phosphorylation of Fyn but not NR2A.**

**Figure C. Src kinase inhibitor and Ketamine cannot inhibit BDNF-induced calcium fluxes**.

**Figure D.** **Anti-GD2 mAb 3F8-induced phosphorylation of Src and NR2B is independent of cAMP-dependent protein kinase (PKA).**

**Figure E. Morphological changes in neuroblastoma cells treated with GD2 ligands**.

**Figure F. Morphological changes in EL4 cells treated with GD2 ligands**.

**Table A**

Relative cellular size (FSC) and complexity (SSC) (10,000 cells analyzed) are increased after treatment with ligands of GD2 ganglioside, in a dose-dependent manner. Cholera toxin-B (binding to GM1 ganglioside) and anti-CD45 mAb yield cell surface binding comparable to 3F8 mAb but do not cause alterations in FSC or SSC.

| **Treatment** | **FSC** | **SSC** |
| --- | --- | --- |
| Untreated | 184 | 163 |
| Mouse IgG | 184 | 163 |
| Anti-GM1 CT-B | 183 | 165 |
| Anti-CD45 | 185 | 162 |
| Anti-GD2 13 nM | 266 | 296 |
| Anti-GD2 130 nM | 295 | 440 |


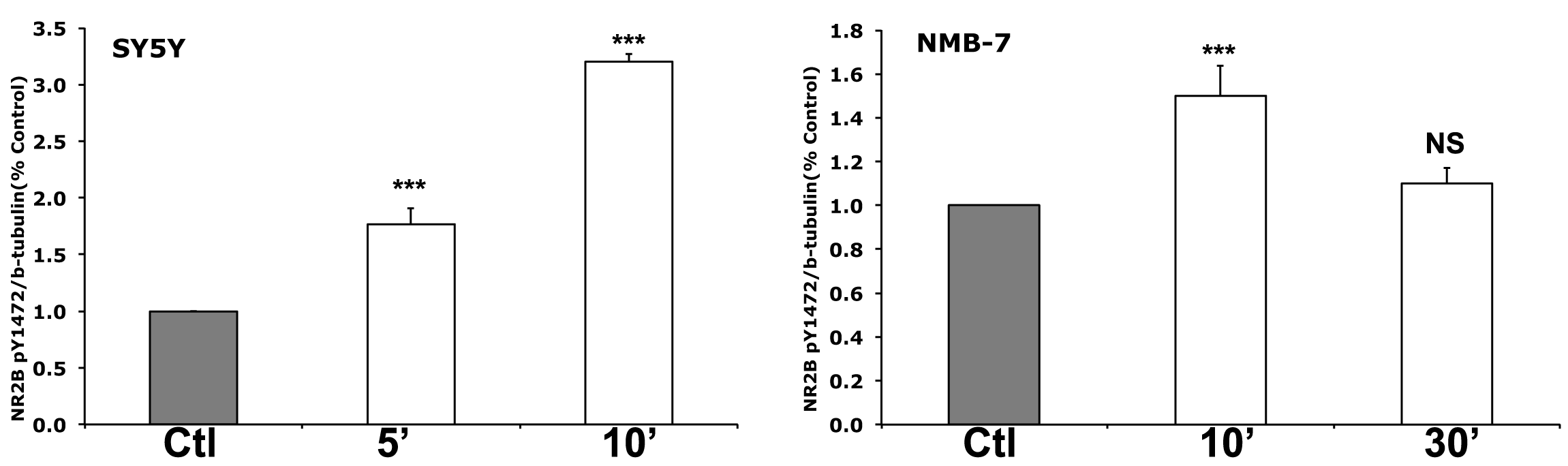


**Figure A.** **Anti-GD2 mAb 3F8 phosphorylates NR2B-Tyr^1472^ in SY5Y and NMB-7 cells.**

SY5Y and NMB-7 cells were treated with mAb 3F8 (10 nM), control mIgG (10 nM) for the indicated times. Western blot analyses of whole cell lysates were done with specific anti-phospho-NR2B pY1472 antibodies. Blot data were standardized to β-tubulin III levels, and were quantified versus untreated cells = 100%, n= 3 independent experiments each in triplicate ± SEM. *** p<0.01 versus control.


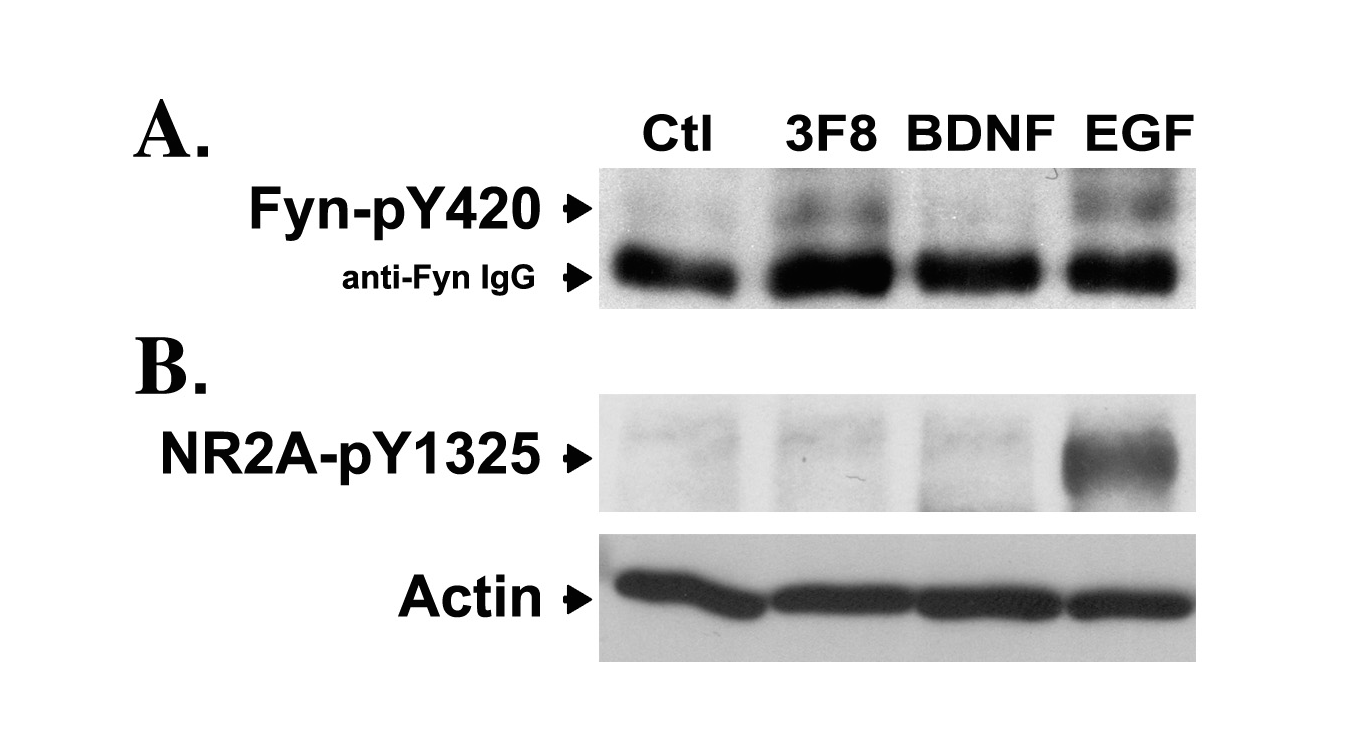


**Figure B.** **Anti-GD2 mAb 3F8 induces the phosphorylation of Fyn but not NR2A.**

SH-SY5Y-TrkB cells were treated with mAb 3F8 (10 nM), control mIgG (10 nM), BDNF (4 nM) or positive control EGF (20 ng/ml) for 10 minutes.

(A) Anti-GD2 antibody 3F8 induces the phosphorylation of Fyn-Tyr^420^. Immunoprecipitation with anti-Fyn antibody from whole cell extracts was followed by Western Blot analyses using a specific anti-phospho-Src Family antibody. EGF treatment is positive control.

(B) Anti-GD2 antibody 3F8 does not induce the phosphorylation of NR2A-Tyr^1325^. Western blot analyses of whole cell lysates were done with specific anti-phospho-NR2A antibodies. EGF treatment is positive control.


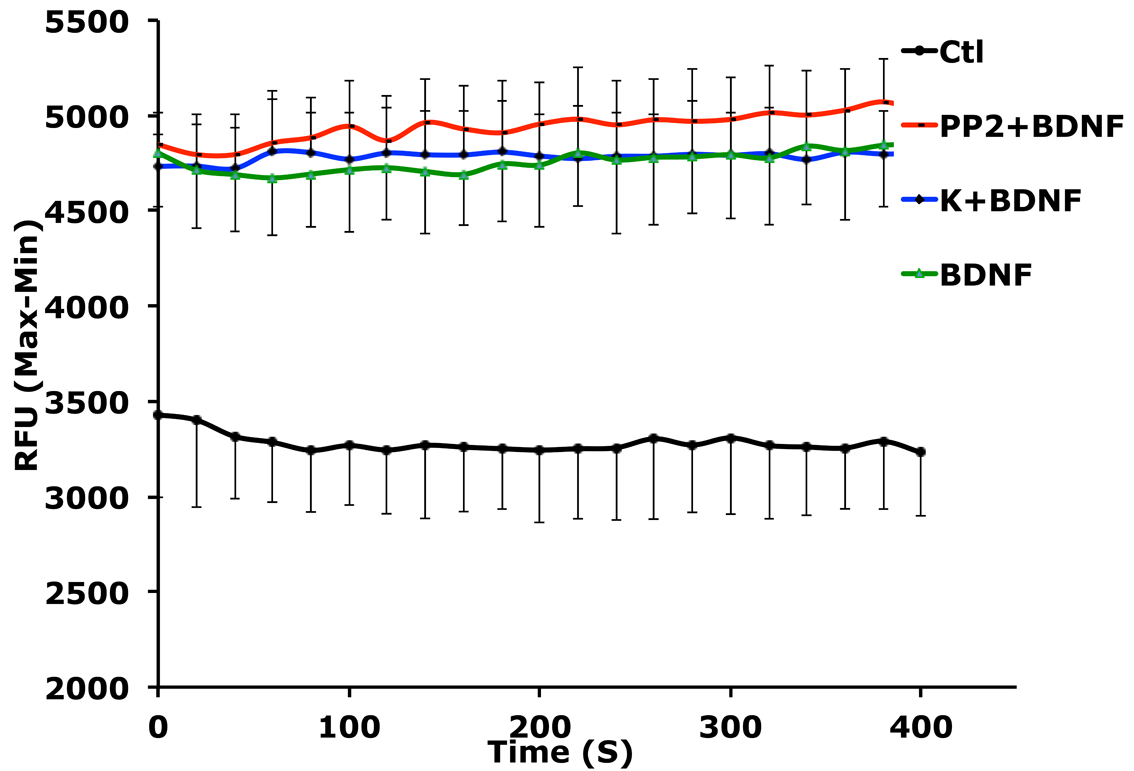


**Figure C. Src kinase inhibitor and Ketamine do not inhibit BDNF-induced calcium fluxes**.

SH-SY5Y-TrkB cells loaded with Fluo-8NW dye solution were treated with BDNF (4 nM). Negative controls buffer or vehicle DMSO were also tested. For pharmacological inhibition, the Src kinase inhibitor PP2 (20 μM) or the NMDA-R antagonist ketamine (20 μM) were added 30 minutes before For Ca^++^ assays, the data are a representative result of three independent experiments each done in quadruplicate ± SD.


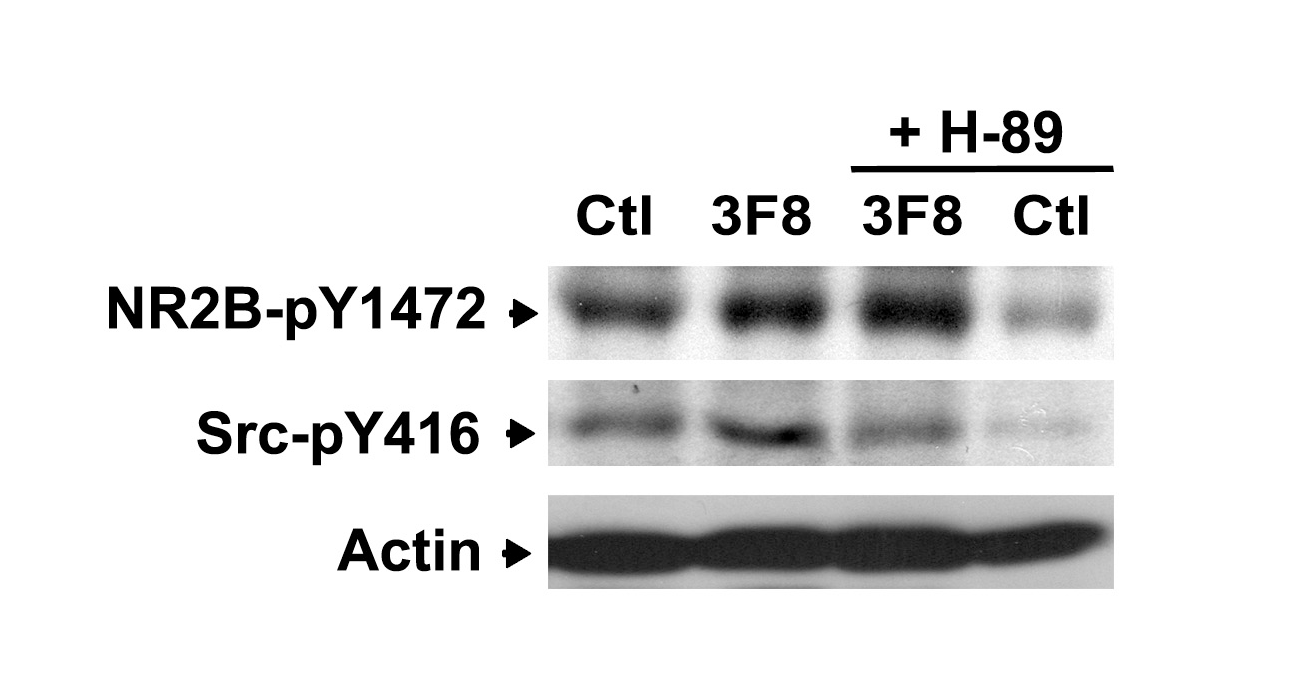


**Figure D.** **Anti-GD2 mAb 3F8-induced phosphorylation of Src and NR2B is independent of cAMP-dependent protein kinase (PKA).**

SH-SY5Y-TrkB cells were pre-treated with vehicle (DMSO) or the cell-permeable PKA inhibitor H-89 (20 μM) for 30 minutes before stimulation with 3F8 (10 nM) or control mIgG (10 nM). H-89 pre-treatment reduced the basal phosphorylation levels but did not prevent anti-GD2 mAb 3F8 induction of Src or NR2B phosphorylation. Western blot analyses of whole cell lysates were done with specific anti-phospho-NR2B or anti-phospho-Src antibodies.


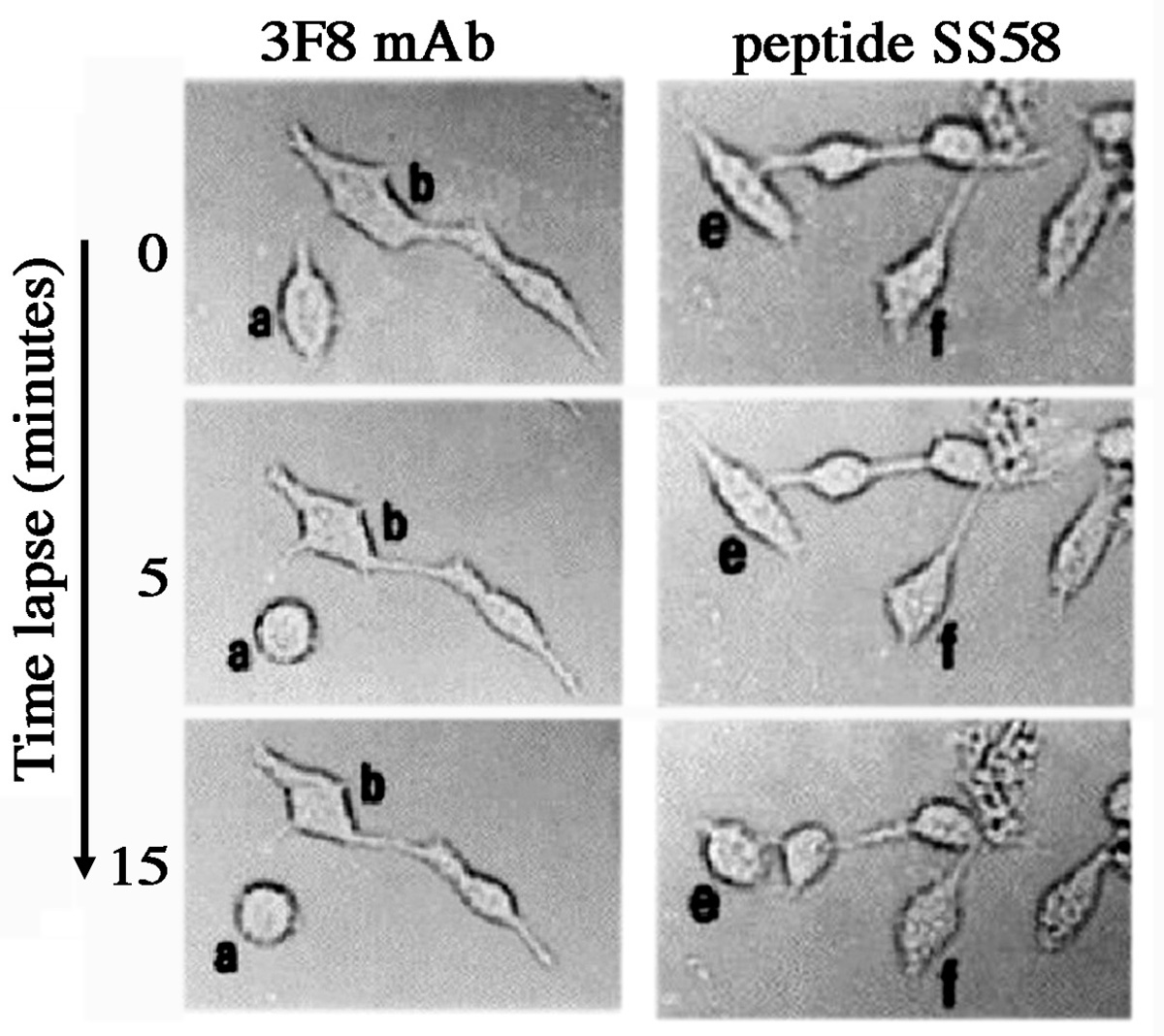


**Figure E. Morphological changes in neuroblastoma cells treated with GD2 ligands**.

Adherent NMB-7 cells grown in complete media were treated with 3F8 (50 nM) or SS58 (10 μM). Time-lapse photographs were taken at 100X magnification at the indicated times of incubation: 0’ (control starting time), 5’, 15’. Cells change in morphology over time. See examples in the change in cells labeled with letters “a”, “b” for mAb 3F8 treatment, and “e”, “f” for SS58 treatment.


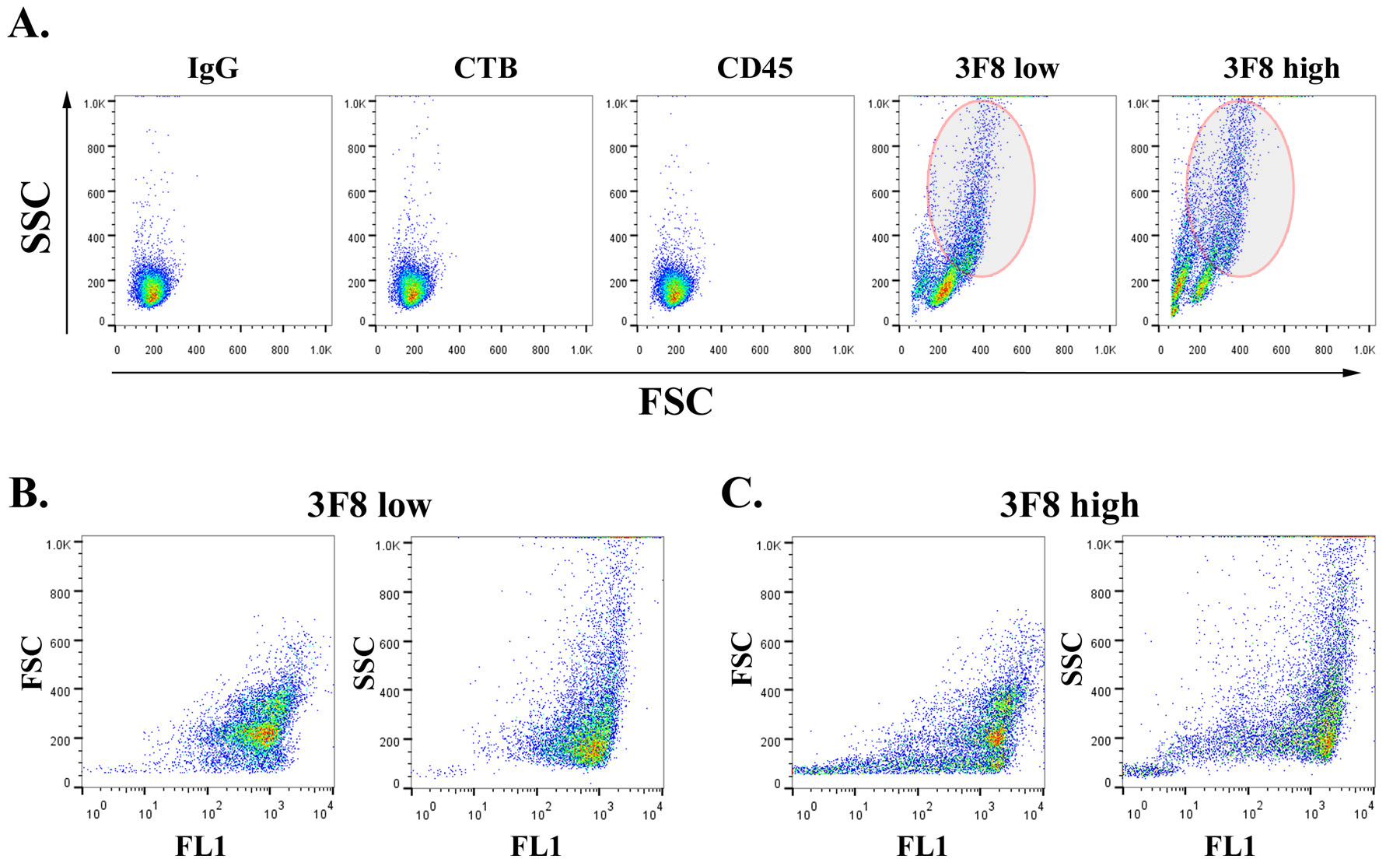


**Figure F. Morphological changes in EL4 cells treated with GD2 ligands**.

Single cell suspensions of EL4 were treated for 15 minutes with the GD2 ligand 3F8 mAb or with controls as indicated, and live cells were freshly analyzed by FACScan.

(**A**) FSC *versus* SSC scatters demonstrating a change in morphology after treatment with GD2 ligands (cells within red ellipse), but not after treatment with controls mouse IgG, cholera-toxin B subunit binding to GM1 ganglioside, or CD45 mAbs. (**B and C**) show FSC *versus* Fluorescence and SSC *versus* Fluorescence scatters for the mAb 3F8-treated cells at 13 nM (median fluorescence 879 units) or 130 nM (median fluorescence 1943 units). Data demonstrate that the cells undergoing the most significant morphological changes are the cells with the highest level of immunostaining.
